# Image and Imaging an Emergency Department: Expense and Benefit of Different Quality Assessment Methods

**DOI:** 10.1155/2013/213263

**Published:** 2013-07-25

**Authors:** Carmen Andrea Pfortmueller, Michael Keller, Urs Mueller, Heinz Zimmermann, Aristomenis Konstantinos Exadaktylos

**Affiliations:** ^1^Department of General Internal Medicine, Bern University Hospital, 3010 Bern, Switzerland; ^2^Emergency Department, Bern University Hospital, 3010 Bern, Switzerland; ^3^Health Care Research Institute AG, Josefstraße 92, 8005 Zurich, Switzerland

## Abstract

*Introduction*. In this era of high-tech medicine, it is becoming increasingly important to assess patient satisfaction. There are several methods to do so, but these differ greatly in terms of cost, time, and labour and external validity. The aim of this study is to describe and compare the structure and implementation of different methods to assess the satisfaction of patients in an emergency department. *Methods*. The structure and implementation of the different methods to assess patient satisfaction were evaluated on the basis of a 90-minute standardised interview. *Results*. We identified a total of six different methods in six different hospitals. The average number of patients assessed was 5012, with a range from 230 (M5) to 20 000 patients (M2). In four methods (M1, M3, M5, and M6), the questionnaire was composed by a specialised external institute. In two methods, the questionnaire was created by the hospital itself (M2, M4).The median response rate was 58.4% (range 9–97.8%). With a reminder, the response rate increased by 60% (M3). *Conclusion*. The ideal method to assess patient satisfaction in the emergency department setting is to use a patient-based, in-emergency department-based assessment of patient satisfaction, planned and guided by expert personnel.

## 1. Introduction

In recent decades, there have been major technical improvements in the health systems of western countries [1]. In this era of high technology, patient satisfaction has become increasingly important [1, 2]. Since the 1950s, patient satisfaction has had an important role in the evaluation of medical care [2].

There are various reasons why it may be profitable for a hospital to perform surveys on patient satisfaction [3]. Several studies have found that satisfied patients suffer less pain [4, 5]; they require fewer (secondary) operations and more rarely have complications [4, 5]. Moreover, satisfied patients exhibit better compliance [4–6]. This simplifies therapy and enhances treatment efficiency [6]. There are also reports that satisfied patients stay for up to 50% less time in hospitals [1, 4, 7]. On the other hand, patient dissatisfaction is a decisive reason for complaints after leaving the hospital [8] as well as for litigation [5, 9]. Thus, an improvement in patient satisfaction can make a major contribution to cost reduction and to maintaining competitiveness [3]. Performing surveys on patient satisfaction is, therefore, an essential patient-centred improvement process [10]. 

A very wide variety of methods are now in use to assess patient satisfaction with some drastic differences in costs, time expenditure, and external validity [1]. Although there have been extensive discussions on patient satisfaction in itself, no comparison has yet been performed between the various conventional methods. In particular, there has been no cost benefit analysis. The objective of the present study is to compare the structure and implementation of different patient satisfaction surveys in emergency wards. 

## 2. Methods

### 2.1. Questionnaire and Interview

On the basis of empirical considerations and after studying the relevant literature, the questionnaire was created by a quality manager, an interview expert, and a clinician with epidemiological training. The standardised interview was performed by a single independent person, who had previously been trained in performing interviews. In each case, the person interviewedwas responsible for patient satisfaction in the corresponding emergency ward. The interview lasted 90 minutes.

### 2.2. Contents of the Questionnaire

The questionnaire covered the method of assessing the patient (mode of assessment, site of assessment, and time point of assessment), the financial expenditure (preparation of the project, commissioning an institute, expenditure per patient, and expenditure for evaluating the data), and the reliability of the data obtained (response and response after reminder). See Table 1.

### 2.3. Selection of Emergency Wards

The emergency wards were randomly selected and contacted in writing. A total of six different assessment methods were recorded. A typical reference hospital was selected for each assessment method. Telephonic assessment was excluded from the study.

### 2.4. Definition of the Costs

All expenses that arise from the organisation and the implementation of the assessment of patient satisfaction were recorded. The costs were given per measurement cycle, as this includes one-off basic costs that are independent of the duration. The costs that arise directly for the individual patient—such as distribution and digitalization—are given per patient, as the number of patients varies greatly between the methods. The costs that could not be given directly during the interviews were estimated together with the interview partner and then validated by a quality expert.  Table 2 shows the different phases of the performance of a patient assessment.

### 2.5. Time Expended

All staff deployment that arose in direct connection with the assessment of patient satisfaction was rated as work performed. If persons outside the hospital were deployed, this was counted as costs. In order to make it possible to assess the costs independently of the country and the currency, the *work unit *(WU) was introduced, where 100 WUs correspond to one hour of work. For the conversion into Swiss Francs, a mean gross hourly wage of CHF 58.- (= 46.40 Euro) was assumed.

## 3. Results

**Figure 1:**
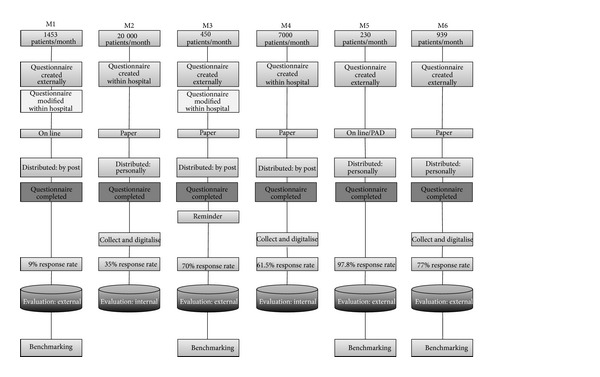
Characterisation of the recorded methods on the assessment of patient satisfaction.

A total of six different methods of assessing patient satisfaction were investigated (Figure 1). On average, 5012 patients per month were interviewed, ranging from 230 patients per month with M5 to 20000 patients per month with M2. All of the questionnaires were similar in length. For four of the methods, an external institute was commissioned to create a questionnaire (M1, M3, M5, and M6); for the other two methods (M2, M4), the questionnaire was created by the corresponding hospital. The hospitals in M1 and M3 commissioned an institute, and also independently created their own questions. For two of the methods, the questionnaire was provided online (M1, M5), but for the other four methods it was printed on paper (M2–M4, M6). For three methods, the assessment was performed at home (M1, M3, and M4) and for the other three on discharge from the hospital. A reminder was only sent for M3. The mean response rate was 58.4% (range 9–97.8%). The reminder (M3) increased the response rate by 60%. For the methods in which the assessment was performed in the hospital, the mean response rate was 69.8%, which is much higher than when the questionnaires were provided at home (46.8%). When the questionnaire was issued at the emergency ward to be completed at home (M2), the response rate was 35%. Invalid questionnaires were more frequent with printed questionnaires than online. In four methods, the recorded data were evaluated externally (M1, M3, M5, and M6), whereas for two methods the evaluation was performed by the corresponding emergency ward (M2, M4). The results from three methods were immediately available (M1, M5, and M6) and those from the other three methods after three months (M2–4). Benchmarking was possible for four methods (M1, M3, M5, and M6), this was always performed by external institutes.

There were differences between the individual methods with respect to both the costs and the time expended, see Table 3. The cheapest method was M5, for which the creation of the questionnaire, the preparation of the infrastructure, and the evaluation of the data were all performed by an external institute. The most expensive was method M2, for which the emergency ward performed the whole patient assessment alone. Commissioning an external institute was markedly less expensive than creating and evaluating the questionnaire internally. The most expensive factor was the creation of the questionnaire. Its creation within the hospital (M2, M4) and its modification (M1, M3) required much more time. 

## 4. Discussion

The present study uses an interview for the description of the advantages and disadvantages of six different methods to assess patient satisfaction. There are marked differences between the individual methods with respect to costs, time expended, and the response rate.

- Findings on the response rate are the following.
  - The response rate is markedly higher if patient satisfaction is assessed in the hospital.
  - Only a few patients will comply with a letter that requests them to complete the questionnaire on discharge from the hospital. Thus, this mode of assessment cannot be recommended.
  - A very good response rate can be recorded by online recording within the hospital, even though this involves additional technical and staff deployment. For example, older patients may require specific support when completing the questionnaire on the computer.
  - If a member of the hospital staff personally enquires about the patient assessment, this raises the response rate. Nevertheless, it must be born in mind that persons participating directly in the treatment may not be involved in the patient assessment, in order to avoid prejudicing the patient's response.
  - It is known from the literature [2], the rate of response is markedly increased by a reminder.
- Costs and resources are the following.
  - Assessments developed and performed by the hospitals themselves require relatively high investment and personnel resources.
  - If an existing questionnaire is modified within the hospital, this requires much more additional time and, therefore, additional costs. Moreover, the hospital staff often lack the expertise needed to implement high-quality data collection and to estimate the resulting costs correctly in advance.
  - It seems to be a cheaper alternative to commission an external assessment institute. This is particularly the case when the price includes not only the questionnaire and its evaluation, but also the infrastructure. Another advantage is that an assessment institute works with a cost budget, which can be clearly assessed by the corresponding hospital.
- Additional points are the following points.
  - Questionnaires from external assessment institutes are mostly validated.
  - External institutes can provide reference values.

## 5. Weaknesses and Strengths of the Study

The present study has the following weaknesses.

-  The resources needed to process the patient assessment forms were not always clearly defined by the hospitals and sometimes had to be estimated. This made it difficult to list the costs and could possibly have led to their underestimation.
-  In many hospitals, the resources linked to the planning of the patient assessment are not precisely documented. It is, therefore, possible that the time expended and the resulting costs were considerably underestimated.
-  Very different numbers of patients were assessed by each method.

In spite of these weaknesses, our data permit relatively clear conclusions. It would be worthwhile to support these by recording prospective data on the effective costs of patient assessments. 

## 6. Conclusion

This study is the first comparison of different methods of performing assessments of patient satisfaction on emergency wards on the basis of examples. It was shown that patient-optimised online assessment performed in the hospital can give a very good response rate. The costs and the time expended can be greatly reduced if an external institute is commissioned. This also offers the possibility of benchmarking. Nevertheless, further prospective studies are needed to validate our results and to deepen our knowledge.

## Figures and Tables

**Table 1:** Summary of the questionnaire used.

	*Methods *
	General organisation and course of the assessment
	Number of persons interviewed/time unit
	Response to questionnaire/time unit
	Site of interview (hospital/at home)
	Reminder
	Incomplete questionnaires
	Assessment period

	*Organisation *
	Process definition
	Material (printed material, stamps, etc.)
	Personnel (number, qualification)
	Infrastructure (computer, tablet, copier, etc.)

	*Cost/time expended *
	Number of persons involved (time expended, involvement, responsibility, etc.)
	Infrastructure (computer, office, printer, etc.)
	Staff training
	External institute
	Per planning phase (see Table 2)

**Table 2:** Overview of the different phases of an assessment of patient satisfaction.

	*Preparation phase *
	Project planning
	Create questionnaire (internal/external)
	Preparation of the infrastructure
	Staff training

	*Measurement period *
	Questionnaire printed or put on line
	Provide questionnaire or access information
	Collect printed questionnaires
	Digitalise printed questionnaires

	*Evaluation phase *
	Data clean-up
	Evaluation (internal/external)
	Evaluation record

**Table 3:** Financial expenditure and time expended.

		M1	M2	M3	M4	M5	M6
Preparation phase	Commissioning an institute	7500.-	0	10000.-	0	1500.-	1500.-
	Creating a questionnaire	14400	10440*	2700	2800	∗∗	∗∗
	Infrastructure	∗∗	9600.-*	∗∗	2500.-*	∗∗	600.-
	Training/communication	400	278.4*	400	6400	0	0

Measurement period	Distribution	1.8	3.3	16	5	3.3	4.2
	Digitalisation	0	7	0	3.3	0	6.7

Evaluation phase	Data clean-up	∗∗	4800	∗∗	2784*	∗∗	∗∗
	Evaluation	∗∗	6000	∗∗	3480*	∗∗	∗∗

Total fixed costs (.-)		7500	28080	10000	10800	1500	2100
Total time expended (WU)		14801.8	10810.3	3116	9208.3	3.3	10.9
Total financial expenditure (.-)		8585.044	6269.974	1807.28	5340.814	1.914	6.322
Total costs		**16085.044**	**34349.974**	**11807.28**	**16140.814**	**1501.914**	**2106.322**

*Values estimated.

**Costs included if institute commissioned.

## References

[1] Ruprecht TM (2000). Qulität quo vadis—die Perspektive der Patienten. *Gesellschaft Für Qualitätsmanagement in Der Gesundheitsversorgung*.

[2] Braun S, Kreimeier S, Greiner W (2010). Measuring patient satisfaction in integrated healthcare projects—a pilot study with a modified ZAP questionnaire. *Zeitschrift fur Evidenz, Fortbildung und Qualitat im Gesundheitswesen*.

[3] Kaderli R, Pfortmuller CA, Businger : AP (2012). Healthcare quality management in Switzerland—a survey among providers. *Swiss Medical Weekly*.

[4] Sobel DS (1995). Rethinking medicine: improving health outcomes with cost-effective psychosocial interventions. *Psychosomatic Medicine*.

[5] Welch SJ (2010). Twenty years of patient satisfaction research applied to the emergency department: a qualitative review. *American Journal of Medical Quality*.

[6] Miaoulis G, Gutman J, Snow MM (2009). Closing the gap: the patient-physician disconnect. *Health Marketing Quarterly*.

[7] Rogner O, Frey D, Havemann D (1984). Psychische Faktoren unfalltraumatologischer Genesungsverlaufe. *Zeitschrift Für Unfallchirurgie, Versicherungsmedizin und Berufskrankheiten*.

[8] Mayer TA, Zimmermann PG (1999). ED customer satisfaction survival skills: one hospital’s experience. *Journal of Emergency Nursing*.

[9] Vincent C, Young M, Phillips A (1994). Why do people sue doctors? A study of patients and relatives taking legal action. *The Lancet*.

[10] Patientenbefragungen: Verfahrensvorschlag. http://www.picker-europe.de/.

